# Peri-Interventional Hemodynamic Management Strategies for Percutaneous Chemosaturation of the Liver in Metastatic Cancer

**DOI:** 10.3390/cancers16213698

**Published:** 2024-11-01

**Authors:** Patrick Rehn, Benjamin Tan, Jan Turra, Patrick Adler, Philipp Mayer, Dania Fischer, Mascha O. Fiedler-Kalenka, Felix C. F. Schmitt, De-Hua Chang, Christoph Lichtenstern, Mark O. Wielpütz, Hans-Ulrich Kauczor, Markus A. Weigand, Maximilian Dietrich

**Affiliations:** 1 Department of Anesthesiology, Heidelberg University Hospital, Im Neuenheimer Feld 420, 69120 Heidelberg, Germany; patrick.rehn@med.uni-heidelberg.de (P.R.);; 2 Department of Pediatric Cardiology and Congenital Heart Diseases, Center for Child and Adolescent Medicine, University Hospital Heidelberg, Im Neuenheimer Feld 430, 69120 Heidelberg, Germany; 3 Department of Perfusion, Heidelberg University Hospital, Im Neuenheimer Feld 420, 69120 Heidelberg, Germany; 4 Department of Radiology, Heidelberg University Hospital, Im Neuenheimer Feld 420, 69120 Heidelberg, Germany; 5 Department of Radiology, Cantonal Hospital of Lucerne, 6000 Lucerne, Switzerland; 6 Translational Lung Research Center (TLRC) Heidelberg, German Center for Lung Research (DZL), Im Neuenheimer Feld 130.3, 69120 Heidelberg, Germany

**Keywords:** chemosaturation, uveal melanoma, anesthesia, shock, hemodynamics, catecholamines

## Abstract

**Background:** Hepatic chemosaturation for inoperable liver tumors is a palliative treatment option with a beneficial effect on survival. However, the procedure regularly leads to circulatory failure during the filtration phase, and hemodynamic management is challenging. Our study aimed to compare two different strategies for hemodynamic management during chemosaturation to develop hypotheses for improving patient care and reducing peri-interventional morbidity. **Methods:** We conducted a single-center retrospective cohort study including 66 procedures of chemosaturation between May 2016 and March 2024. Procedures were divided into two groups: group 1 was managed with norepinephrine as the only vasopressor and liberal use of hydroxyethyl starch (HES). Group 2 was managed with norepinephrine and vasopressin and the preferred use of balanced crystalloids. We compared these two groups with respect to hemodynamic parameters, laboratory values, and post-interventional complications. **Results:** The heart rate was highest and the mean arterial pressure (MAP) was lowest during the filtration phase in both groups (*p* = 0.868, *p* = 0.270). The vasoactive inotropic score (VIS) was significantly higher in group 2 during the filtration phase (31.5 vs. 89, *p* < 0.001). Group 1 received significantly more HES overall (1000 mL vs. 0 mL, *p* < 0.001). Lactate levels at admission to the ICU were higher in group 1 (22.9 vs. 14.45 mg/dL, *p* = 0.041). Platelet counts were lower in group 2 from directly after chemosaturation through day 2 (*p* = 0.022, *p* = 0.001, *p* = 0.032). The INR differed significantly directly after chemosaturation (1.13 vs. 1.26, *p* = 0.015). Overall, group 1 received significantly more blood products peri-interventionally. There were two bleedings and one ischemic stroke in the overall cohort. There was no peri-interventional mortality. **Conclusions:** Advanced hemodynamic management ensures low peri-interventional mortality and morbidity. High-dose vasopressors, including vasopressin and the preferred use of balanced crystalloids, are sufficient to stabilize circulatory function during chemosaturation.

## Simple Summary

Chemosaturation for inoperable liver tumors can improve survival but often causes circulatory problems during filtration, making management challenging. In a study of 66 cases from 2016 to 2024, two approaches were compared: group 1 used one type of vasoactive drug (norepinephrine) and colloids, while group 2 used two types of vasoactive drugs (norepinephrine, vasopressin) and balanced crystalloids instead of colloids. Group 2 showed higher heart rates and lower blood pressure during filtration, with a higher dose of vasoactive drugs. Group 1 had higher lactate levels and used more blood products, while group 2 showed lower platelet counts. Both approaches showed no peri-interventional mortality. High-dose vasoactive drug use including vasopressin and balanced crystalloids is sufficient to stabilize circulatory function during chemosaturation.

## 1. Background

Hepatic chemosaturation with melphalan allows high doses of chemotherapy to be delivered directly to the liver while minimizing systemic exposure and toxicity, making it a valuable palliative treatment option for patients with inoperable liver tumors [1]. Several studies reported a beneficial effect on survival in various tumor entities [2,3,4,5]. The system consists of intravascular catheters and an extracorporeal circulation (ECC) with an active-carbon filter for hemofiltration. The chemotherapeutic agent of choice is melphalan hydrochloride. It proved to be effective as it has demonstrated both high efficacy in the treatment of primary liver tumors and a variety of liver metastases of different origins, as well as reversible liver toxicity [6,7,8,9,10]. Chemosaturation is performed under general anesthesia. The chemotherapeutic agent is applied directly to the branches of the hepatic artery, supplying the tumor via a microcatheter through a left femoral access. A double balloon catheter for hepatic vein isolation is inserted through a right femoral access. The cephalad balloon is placed at the junction of the inferior vena cava (VCI) and the right atrium (RA). The caudal balloon is placed infrahepatically in the VCI so that the venous outflow from the hepatic veins is isolated between the two balloons. The hepatic venous blood, which is fully saturated with melphalan, is aspirated through the double balloon catheter via the ECC. In the ECC system, the blood passes through two active-carbon filters, whereby the chemotherapeutic agent is filtered out of the blood. The purified blood is then returned to the patient’s systemic circulation via the right internal jugular vein (IJV) [5].

The initiation of the activated charcoal filter leads to a massive drop in blood pressure and massive hemodynamic instability during the filtration phase [11,12,13]. Various theories are discussed, which assume either catecholamine filtration, a systemic inflammatory reaction with cytokine release, or a release or upregulated synthesis of nitric oxide (NO) [5]. Interestingly, McEwan et al. found a decrease in catecholamine levels post-filter in an experimental study in swine [14]. Correspondingly, the observed hemodynamic changes match those seen in distributive shock. There are several approaches to manage this, including fluid therapy and the calculated or differentiated administration of catecholamines. To our knowledge, there are currently no human studies available investigating the exact cause of the rapid hemodynamic deterioration. Therefore, the intervention is considered a high-risk procedure for anesthetic management and is only performed in a few specialized centers. To date, no experimental data concerning the ideal hemodynamic management in this highly selected cohort are available. Another transient effect of chemosaturation observed in almost every patient is the alteration of coagulation parameters post-intervention [5]. It is unclear whether pharmacological interventions are necessary.

The aim of this single-center retrospective cohort study was to compare two different peri-interventional strategies for hemodynamic treatment based on either norepinephrine alone or in combination with vasopressin in order to develop hypotheses for improving patient care and reducing peri-interventional morbidity.

## 2. Methods

### 2.1. Study Population and Data Collection

All patients who underwent percutaneous chemosaturation of the liver at University Hospital Heidelberg, Department of Diagnostic and Interventional Radiology from May 2016 to March 2024 were included. According to local standards, patients with severe cardiac, pulmonal, hepatic, or renal comorbidities were not eligible to receive chemosaturation. Indication for treatment was determined by an interdisciplinary tumor board. All patient data were collected retrospectively by manual data extraction from electronic medical records. The extraction included epidemiological data, indication for chemosaturation, comorbidities, laboratory values, catecholamine dose, intra- and post-interventional complications, amount and type of fluid given intra-interventionally, peri-interventional transfusion of blood or blood products, and survival data. Laboratory values were recorded one day before chemosaturation, immediately after patients arrived in the ICU, after chemosaturation, and 1 and 2 days after chemosaturation, respectively. Catecholamine dose was recorded as a continuous flow rate before, during, and after the filtration phase of chemosaturation and at the time of arrival at the ICU. During the given timepoints of chemosaturation, the highest catecholamine flow rate was recorded in each procedure. For MAP, we recorded the lowest value, and for HR, the highest value for each timepoint. We excluded 5 chemosaturation procedures due to the lack of data in the electronic medical report (ISH med, SAP^®^ (Walldorf, Germany) and COPRA PDMS, Copra System GmbH^®^ (Berlin, Germany)).

### 2.2. Procedure

First, patients were placed under general anesthesia using induction agents Propofol (Fresenius Kabi GmbH^®^ (Bad Homburg, Germany)), Sufentanil (Hameln Pharma GmbH^®^ (Hameln, Germany)), and Rocuronium (B. Braun SE^®^ (Melsungen, Germany)). An endotracheal tube was then inserted. General anesthesia was maintained using a balanced approach with Sevoflurane (AbbVie GmbH & Co. KG^®^ (Wiesbaden, Germany)) and a continuous infusion of Remifentanil (Hameln Pharma GmbH^®^) in all patients. A central venous line and an arterial line were placed in every patient. Prior to the start of the procedure, every patient was given peri-interventional antibiotic prophylaxis using 1.5 g Cefuroxim as a single shot. Patent foramen ovale was ruled out by transesophageal echocardiography (TEE). The chemosaturation procedure was done by two interventional radiologists using the CHEMOSAT^®^-Catheter and CHEMOSAT^®^-filtration system (Delcath Systems, Inc., New York, NY, USA) using melphalan as the chemotherapeutic agent. After canulation of the left femoral artery and the right femoral vein, a bolus of 400 IU/kg heparin was administered to achieve an Activated Clotting Time (ACT) > 450 s. If the ACT was <450 s, a second bolus of 100–200 IU/kg heparin was administered, and the ACT was checked again. Filtration was performed by using ECC operated by a perfusionist. Hemodynamic stabilization during the procedure was done by using fluid boluses of either hydroxyethyl starch (HES) solution or balanced crystalloid solution, continuous administration of norepinephrine and vasopressin, and boluses of norepinephrine as needed. The reversal of the heparin’s effect after ECC was done by peripheral infusion of Protamine in a 1:1 ratio of Protamine to heparin with a fixed flow rate of 200 mL/h. After warnings of possible side effects, the Protamine regimen was changed to a dose of 200 IU/kg, also with a fixed flow rate of 200 mL/h and a goal of ACT < 160 s. After extubation, patients were transferred to an intermediate or intensive care unit, where they were monitored for at least 1 night.

Notably, there was a change in peri-interventional anesthesiologic management in June 2018. Because of the possible mechanism of filtration of catecholamines and new evidence by McEwan et al., a more vasopressor-heavy strategy (norepinephrine plus vasopressin) was implemented [14]. Vasopressin (AOP Orphan Pharmaceuticals^®^ (Vienna, Austria)) was added as an adjunct vasopressor, and the use of HES was discontinued. Also, blood products (Prothrombin Complex Concentrate (PCC), Antithrombin-3 (AT3), fibrinogen) were given more restrictively.

### 2.3. Study Design

We divided our retrospective cohort into different subgroups according to the change in the standard operating procedure (SOP) in June 2018. There was strict adherence to the SOP in each group.

Group 1 (NE only) consisted of 16 procedures (in 7 patients). In this group, only noradrenalin was used as a vasopressor in all procedures. HES was given in 75% of the procedures (12 total procedures) as a fluid bolus during the phase of hemodynamic instability. Also, blood products (PCC, fibrinogen, and AT3) were given in 81.3% of the procedures (13 total procedures) to augment deranged coagulation parameters. Doses of these blood products varied and were decided individually by the treating anesthesiologist.

Group 2 (NE + Vaso) consisted of 50 procedures (in 28 patients). Here, noradrenalin and vasopressin were used as vasopressors in all procedures. Fluid blouses were mainly given in the form of balanced crystalloids, and 4% of procedures (2 total procedures) received HES additionally. Moreover, blood products (PCC, fibrinogen, and AT3) were given in 4% of procedures (2 total procedures).

We compared these two groups with respect to hemodynamic parameters, laboratory values, and post-interventional complications. We used the vasoactive inotropic score (VIS) to better compare the hemodynamics and catecholamine doses. The VIS is calculated by the following formula: dopamine dose (μg/kg/min) + dobutamine dose (μg/kg/min) + 100 × epinephrine dose (μg/kg/min) + 100 × norepinephrine dose (μg/kg/min) + 10,000 × vasopressin dose (U/kg/min) + 10 × milrinone dose (μg/kg/min), according to Gaies et al. [15,16].

### 2.4. Ethics

Ethical approval for this study (reference number S314/2022) was provided by the Ethical Committee of the Medical Faculty of Heidelberg University, Alte Glockengießerei 11/1, 69115 Heidelberg, Germany (Chairperson Prof. Dr. med. Dr. h.c. Thomas Strowitzki) on 22 May 2024.

### 2.5. Statistical Methods

Descriptive statistics was performed on all relevant data. Median, interquartile range (IQR), and 95% confidence intervals (CI) for the median, min, and max values were calculated. A comparison of metric values between groups was performed with a Mann–Whitney U Test, and a comparison of change over time was analyzed with a Friedman Test. Categorical values were analyzed using a Chi-Squared Test. The level of significance was set at *p* < 0.05. Metric parameters are reported as median and IQR; categorical parameters are reported as absolute and relative frequencies. Statistical analysis was performed with SPSS version 25.0 (IBM^®^ (Armonk, NY, USA)).

## 3. Results

### 3.1. Indication for Chemosaturation and Baseline Characteristics

A total of 66 chemosaturation procedures in 35 individual patients were analyzed. Indications for chemosaturation were predominantly metastatic choroidal melanoma (50 of 66 total procedures (75.8%), 23 of 35 patients (65.7%)) and other uveal melanomas (11 of 66 total procedures (16.7%), 8 of 35 patients (22.9%)). Other indications included metastatic melanoma of the skin (1 total; 1,5%), cholangiocarcinoma (3 of 66 total procedures (4.5%), 2 of 35 patients (5.7%)) and pancreatoblastoma (1 total; 1.5%).

The baseline patient characteristics are described in Table 1. The median age was 58 (IQR 52–63) overall: 47 (IQR 35–56) in group 1 and 59 (IQR 55–66) in group 2. The difference between groups 1 and 2 was significant (*p* < 0.001). Sex, BMI, and the duration of the procedure did not vary significantly between the groups. No relevant comorbidities (coronary artery disease (CAD), arterial hypertension, renal insufficiency, and diabetes) were recorded in group 1, whereas in group 2, 4% had CAD, 34% had arterial hypertension, and 8% had diabetes. Only the difference in arterial hypertension reached statistical significance (*p* = 0.007).

### 3.2. Hemodynamic Parameters

**Figure 1:**
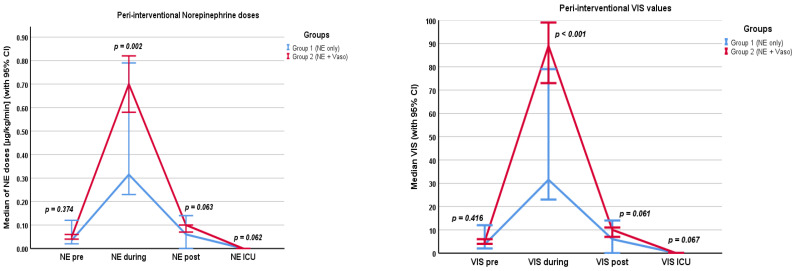
Norepinephrine doses (in µg/kg/min) and VIS values pre/during/post filtration phase and at admission to the ICU. NE: norepinephrine, Vaso: vasopressin, VIS: vasoactive inotropic score, CI: confidence interval.

**Figure 2:**
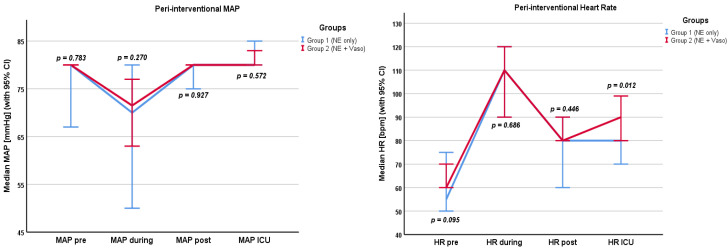
MAP (in mmHg) and heart rate (in bpm) pre/during/post filtration phase and at admission to the ICU. MAP: mean arterial pressure, HR: heart rate, NE: norepinephrine, Vaso: vasopressin, CI: confidence interval.

**Figure 3:**
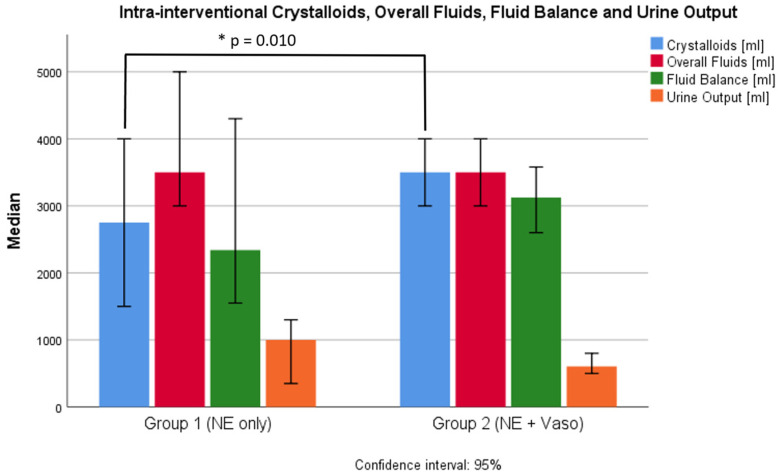
Crystalloids, overall fluids, fluid balance, and urine output intra-interventionally. NE: norepinephrine, Vaso: vasopressin, CI: confidence interval, *: significant difference.

Hemodynamic parameters are shown in the Supplementary Materials (Table S1) and in Figure 1, Figure 2 and Figure 3. Values were recorded pre/during/post the filtration phase and at admission to the ICU. Crystalloids, overall fluids, HES, and urine output were only recorded intra-interventionally.

#### 3.2.1. Whole Cohort

Norepinephrine levels were the highest during the filtration phase (0.6 µg/kg/min, IQR 0.36–1.0 µg/kg/min), and most patients did not need any catecholamines at admission to the ICU (0.00 µg/kg/min, IQR 0.00–0.01 µg/kg/min). Accordingly, the VIS was highest during the filtration phase (78.5, IQR 46.6–108). Patients received a median of 3500 mL (IQR 3000–4000 mL) of crystalloids. The median fluid balance after chemosaturation was +2950 mL (IQR +2300–3988.75 mL) and the median urine output was 700 mL (IQR 450–1010 mL). During the filtration phase, the median heart rate was highest (105 bpm, IQR 90–120 bpm) and the median MAP was lowest (73 mmHg, IQR 60–81 mmHg).

#### 3.2.2. Groups

Differences in catecholamine dosages pre and post the filtration phase and at the time of admission to the ICU were not significant between the groups. Despite additional usage of vasopressin in group 2, the norepinephrine dose in group 2 (0.71 µg/kg/min, IQR 0.2–1.0 µg/kg/min) was higher than in group 1 (0.32 µg/kg/min, IQR 0.25–0.72 µg/kg/min) during the filtration phase (*p* = 0.002). The VIS was correspondingly higher in group 2 (89, IQR 66.5–114.2) than in group 1 (31.5, IQR 24.5–72) during the filtration phase (*p* < 0.001). The VIS did not differ significantly before and after filtration or at the time of admission to the ICU. Also, the volume of crystalloids given in group 1 (2750 mL, IQR 2000–3875 mL) was lower than in group 2 (3500 mL, IQR 3000–4500 mL). This difference was significant (*p* = 0.01). Group 1 received significantly more HES (1000 mL, IQR 250–1500 mL) than group 2 (*p* < 0.001). Here, HES was given in two procedures as a single bolus of 500 mL per procedure. The overall fluid balance did not differ significantly between the groups (*p* = 0.097). The overall fluid input of crystalloids and colloids was 3500 mL (IQR 3000–4375 mL) in group 1 and 3500 mL (IQR 3000–4500 mL) in group 2 (*p* = 0.698). Peri-interventional urine output also did not differ significantly between the groups (*p* = 0.161). The heart rate at admission to the ICU was higher in group 2 (90 bpm, IQR 77–100 bpm) than in group 1 (80 bpm, IQR 70–90 bpm) (*p* = 0.012).

### 3.3. Laboratory Parameters

**Figure 4:**
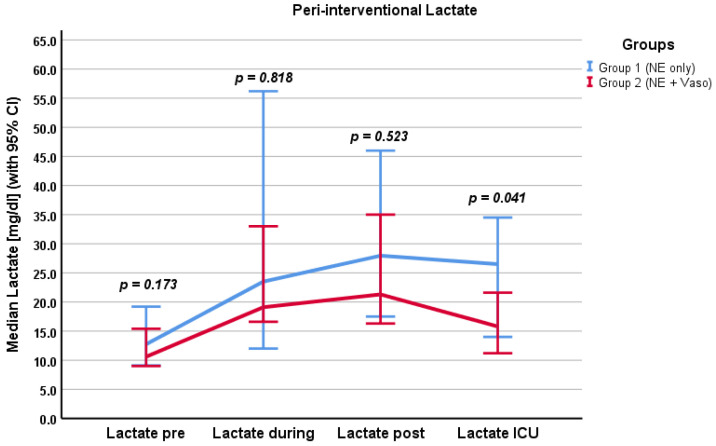
Peri-interventional lactate levels, pre/during/post filtration phase and at admission to the ICU. Values in mg/dL. NE: norepinephrine, Vaso: vasopressin, CI: confidence interval.

**Figure 5:**
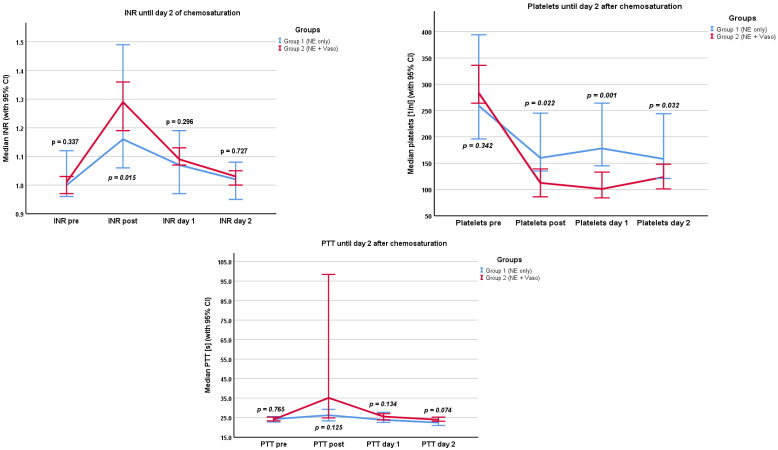
Coagulation parameters pre/post chemosaturation and on day 1 and day 2, respectively, after chemosaturation. PTT in seconds, platelets in 1/nL. NE: norepinephrine, Vaso: vasopressin, CI: confidence interval.

We examined different laboratory parameters relevant to microcirculation, organ damage, and coagulation. The results are presented in the Supplementary Materials (Table S2) and in Figure 4 and Figure 5. Values were recorded before and after chemosaturation and at day 1 and day 2 after chemosaturation, respectively. Lactate levels were recorded pre/during/post the filtration phase and at admission to the ICU.

#### 3.3.1. Whole Cohort

Regarding the whole cohort, lactate showed a tendency to rise until after the filtration phase (*p* < 0.001) and fell until admission to the ICU (*p* < 0.001). Creatinine levels were not significantly higher at day 2 after chemosaturation (*p* = 0.055). Transaminases showed a significant but overall moderate increase until day 2 after chemosaturation (*p* < 0.001). LDH increased significantly until day 2 (*p* < 0.001). PTT and INR levels were initially (after chemosaturation) increased (24 s, IQR 22.6–25.5 s; 1.23, IQR 1.16–1.38) but normalized by day 2 (*p* < 0.001). There was a significant drop in platelets after chemosaturation (median: 284/nl pre to 127/nl post chemosaturation; *p* < 0.001). Platelets remained at this level until day 2 (*p* = 0.328). Hemoglobin fell until day 2 (*p* < 0.001); the nadir level of hemoglobin by day 2 was 6.7 g/dL.

#### 3.3.2. Groups

For Creatinine and LDH, no significant difference between the two groups was found at any timepoint (Creatinine: *p* = 0.805, *p* = 0.410, *p* = 0.742, *p* = 0.916 and LDH: *p* = 0.419, *p* = 0.962, *p* = 0.876, *p* = 0.478). Lactate levels differed significantly between the groups at admission to the ICU (*p* = 0.041). Group 1 had higher lactate levels (22.9 mg/dL, IQR 13.65–31.55 mg/dL) than group 2 (14.45 mg/dL, IQR 11.2–21.3 mg/dL). The MELD Score differed significantly between the two groups post chemosaturation. Group 1 had a lower MELD Score (6, IQR 6–8) than group 2 (8, IQR 7–10), *p* = 0.001. There was no significant difference between the groups in transaminases (GOT and GPT). Platelet count differed between the groups post chemosaturation. Group 1 had a higher platelet count (159, IQR 131–242) than group 2 (113, IQR 69–15; *p* = 0.022). This difference persisted through day 1 (*p* = 0.001) and day 2 (*p* = 0.032). The INR differed significantly directly post chemosaturation (*p* = 0.015). Group 1 had lower INR values (1.13, IQR 1.07–1.34) than group 2 (1.26, IQR 1.18–1.39). This difference did not persist through day 1 (*p* = 0.298) and day 2 (*p* = 0.727). There was no significant difference in PTT between the two groups. Fibrinogen levels were only analyzed post chemosaturation. There was no difference between groups 1 and 2 (*p* = 0.892).

### 3.4. Transfusion of Blood Products

The use of different blood products or blood compounds peri-interventionally (within the day of chemosaturation) was examined. The results are presented in Table 2.

#### 3.4.1. Whole Cohort

Patients received a median of 20 g of albumin (IQR 0–32.5 g). As only patients in group 1 received PCC and most of the AT3 was given in group 1, the median of the PCC for the overall cohort was 0IE (IQR 0–0IE), and the median of AT3 for the overall cohort was also 0IE (IQR 0–0IE). In total, 11 units of RBCs were transfused in seven individual patients. Regarding FFP, eight total units were transfused in two patients. A total of 20 units of platelets were transfused in 10 individual patients.

#### 3.4.2. Groups

More blood products were used in group 1. PCC and fibrinogen were only used in group 1, and AT3 was mostly used in group 1. Correspondingly, there was a statistically significant difference in the administration of PCC, AT3, and fibrinogen between groups 1 and 2 (*p* < 0.001). There was no statistically significant difference in the transfusion of RBCs or FFP between the groups. In group 1, five units of RBCs were transfused in two individual patients and four units of FFP were transfused in one patient. In group 2, six units of RBCs were transfused in five individual patients and four units of FFP were transfused in one patient. There was a statistically significant difference in platelet transfusion (*p* < 0.001). In group 1, a total of 19 units of platelets (median 1.5, IQR 0–2) were transfused in nine individual patients; in group 2, only one unit of platelets was transfused in total.

### 3.5. Complications

Post-interventional complications are presented in Table 3. Overall, three clinically significant complications were recorded (two clinically significant bleedings and one ischemic stroke). Clinically significant bleeding was defined as requiring the following interventions: transfusion of RBCs or other blood products and prolonged stay at the ICU or intermediate care unit. All complications were recorded in group 2. The differences were not statistically significant between the two groups. There was no peri-interventional ICU mortality.

## 4. Discussion

Chemosaturation poses significant anesthesiologic challenges due to its complex procedure and associated hemodynamic instability. This is the first study investigating two different hemodynamic management strategies in hepatic chemosaturation for managing inoperable primary liver tumors and liver metastases.

We found that the VIS was highest during the filtration and rapidly lowered until admission to the ICU in the whole cohort. Moreover, the VIS was higher in group 2 than in group 1 during the filtration phase. However, there was no significant difference in MAP or lactate levels between the groups during or post the filtration phase. In group 1, more blood products were used. Overall, peri-interventional complications were very low but were only registered in group 2.

Our results suggest that a vasopressor-heavy strategy in which vasopressin is used as an adjunct vasopressor and blood products are used more restrictively could be a viable option for anesthesiologic management peri-interventionally. Therefore, we hypothesize that the outlined peri-interventional management of group 2 could lead to improved patient outcomes. However, with the presented results, we cannot demonstrate the superiority or inferiority of either strategy due to the small number of included patients and procedures. Even in a big tertiary center such as the study site, chemosaturation is a rare intervention as patients are highly selected. Nevertheless, this study aimed to formulate hypotheses that require further investigation, as there are currently limited data available.

The exact pathophysiological mechanism behind the pronounced hemodynamic instability during the filtration phase has not yet been finally clarified [5]. A reasonable explanation could be the filtration of endogenous catecholamines by the active-carbon filters of the chemosaturation system. Supporting this theory, McEwan et al. found decreased catecholamine levels post-filtering in an experimental study in swine [14]. The approach of adding vasopressin to norepinephrine rather than colloids to stabilize hemodynamics during the filtration phase could, therefore, be a reasonable choice for rapid stabilization. Patients in group 2 had significantly higher VIS values during chemosaturation and did not receive HES as a colloid.

Chiu et al. observed that giving less fluids and more vasopressors during non-cardiac surgery increased the rate of acute kidney failure [17]. Despite a similar approach in group 2, we did not observe a higher incidence of acute kidney injury in group 2. Creatinine levels post chemosaturation did not significantly differ between the groups, and we did not record any acute kidney injuries in the overall cohort. Also, the intra-interventional urine output was similar in both groups. Because Chiu et al. investigated patients undergoing a broad spectrum of non-cardiac surgeries with potentially more volume loss with respect to blood loss, comparability might be limited. Nevertheless, a possible explanation for the missing link between higher catecholamine doses and acute kidney injury could lie in the mechanism of circulatory failure. As different authors suggested, the filtration of endogenous catecholamines through the active-carbon filter of the chemosaturation system could lead to vasodilatory shock [5,14]. In this context, catecholamine therapy merely replaces the endogenous catecholamines that were lost due to filtration. To definitely answer the question of whether a vasopressor-heavy strategy is non-inferior in this context, prospective studies with larger sample sizes are needed.

At the time of admission to the ICU, the lactate levels of group 2 were lower than in group 1, suggesting a faster lactate clearance. Lactate levels are thought to represent the degree of malperfusion of tissue and are, therefore, assumed to be a marker of microcirculation [18,19]. In distributive shock forms such as septic shock, guidelines recommend the measurement of lactate to guide therapy [20]. Faster lactate clearance was found to correspond with better survival in distributive shock [21]. The observed hemodynamic instability during the filtration phase closely resembles distributive shock. Despite using higher doses of vasopressors and a correspondingly higher VIS in group 2, lactate levels during and after the filtration phase did not differ between the groups. It can therefore be hypothesized that a vasopressor-heavy strategy, including vasopressin for hemodynamic stabilization during the filtration phase, restores adequate tissue perfusion and does not have a detrimental effect on microcirculation. However, lactate is currently called into doubt as being the optimal measurement of microcirculation. The ANDROMEDA-Shock trial did not find better survival for a lactate-guided resuscitation strategy [22]. Even the positive effects of lactate are discussed. In a rat model, lactate improved circulatory function [23]. However, interpretation of lactate levels is often difficult in multimorbid patients with hepatic and renal dysfunction, as lactate is cleared via these pathways [24]. In our cohort, we mainly studied patients without relevant liver or kidney insufficiency or other underlying severe comorbidities.

Overall, there were two clinically significant bleeding events that were defined as events that led to a prolonged stay in the ICU or a need for transfusion. All these events were recorded in group 2. Due to the small number of total events, no statistical significance could be shown. The liberal administration of blood products can be harmful due to possible transfusion-related complications such as transfusion-related lung injury (TRALI) or transfusion-associated circulatory overload (TACO) [25,26]. However, as all bleeding events were recorded in group 2, patients might have benefitted from earlier administration of blood products. Regarding coagulation parameters, there was no difference between the groups except for platelet count. Platelets were significantly lower in group 2. A possible explanation could be that vasopressin, which was administered in high doses (median of 0.0013 U/kg/min), caused aggregation of platelets via the V1 receptor [27,28]. Notably, group 1 received significantly more units of platelets (19 in total vs. 1 in total), which also explains the difference in platelet count. Deranged coagulation parameters normalized in both groups by day 2. However, INR directly post chemosaturation was significantly higher in group 2 but normalized by day 1. As group 1 received significantly more PCC, this difference is well explained. There was no significant difference between the groups by day 1.

Our study has several limitations. Firstly, the small number of patients included is the main weakness of this trial. The incidence of uveal melanoma is approximately six per million in central Europe [29,30]. Of these patients, only about 50% develop metastatic disease [29]. To be eligible for chemosaturation of the liver, these patients must otherwise be healthy as the hemodynamic instability can be drastic. It is, therefore, very difficult to generate higher patient numbers. To overcome this shortcoming, future studies should be conducted in a multicentric manner. Furthermore, the retrospective design can account for possible confounders. Lastly, we did not observe long-term outcome parameters such as 90-day survival or kidney function. Different peri-interventional management strategies could influence long-term outcomes.

## 5. Conclusions

In conclusion, we found that there is considerable peri-interventional hemodynamic instability. Especially during the filtration period of chemosaturation, patients needed high doses of catecholamines to maintain adequate MAP. To account for these profound and dynamic alterations of the hemodynamic status, it is paramount to have an adequate peri-interventional anesthesiologic management strategy. Our findings suggest that supplementing any loss of endogenous catecholamines with norepinephrine and vasopressin, along with a personalized balanced crystalloid fluid therapy, could provide a viable solution. Given the complexity of this procedure, chemosaturation should be conducted only at centers with specialized expertise and comprehensive training for anesthesiologists. We believe that forming dedicated teams of specially trained anesthesiologists is critical for maintaining high standards in this unique intervention. Finally, further prospective studies with larger patient populations are warranted to refine and optimize future treatment strategies.

## Figures and Tables

**Table 1:** Baseline characteristics: data are expressed as median (interquartile range) or as absolute and relative frequencies. BMI: Body Mass index, CAD: coronary artery disease.

Characteristic	Overall n = 66 Procedures	Group 1 (NE Only) n = 16 Procedures	Group 2 (NE + Vaso) n = 50 Procedures	*p* Value
Sex, female	39 (59.1%)	8 (50%)	31 (62%)	0.399
Age, median [y]	58 (52–63)	47 (35–56)	59 (55–66)	<0.001
BMI, median [kg/m^2^]	24.3 (22.2–26.5)	24.3 (19.2–26.8)	23.9 (22.5–26.5)	0.664
Comorbidities				
CAD	2 (3%)	0 (0%)	2 (4%)	0.420
arterial hypertension	17 (25.8%)	0 (0%)	17 (34%)	0.007
renal insufficiency	0 (0%)	0 (0%)	0 (0%)	1
diabetes	4 (6.1%)	0 (0%)	4 (8%)	0.247
Duration of procedure [min]	180 (165–213)	180 (151–210)	195 (165–225)	0.112

**Table 2:** Transfusion of blood products. Data are expressed as median (interquartile range) or total number. NE: norepinephrine, Vaso: vasopressin.

	Overall n = 66 Procedures	Group 1 (NE Only) n = 16 Procedures	Group 2 (NE + Vaso) n = 50 Procedures	*p* Value
Albumin [g]	20 (0–32.5)	0 (0–0)	20 (12.5–40)	<0.001
PCC [IE]	0 (0–0)	2500 (250–3000)	0 (0–0)	<0.001
RBCs [units, total]	11	5	6	0.470
Platelets [units]	0 (0–0)	1.5 (0–2); 19 (total)	0 (0–0); 1 (total)	<0.001
FFP [units, total]	8	4	4	0.392
AT3 [IE]	0 (0–0)	1000 (1000–1000)	0 (0–0)	<0.001
Fibrinogen [g]	0 (0–0)	1.5 (0–4)	0 (0–0)	<0.001

**Table 3:** Post-interventional complications. Data are expressed as total number (%). NE: norepinephrine, Vaso: vasopressin.

	Overall n = 66 Procedures	Group 1 (NE Only) n = 16 Procedures	Group 2 (NE + Vaso) n = 50 Procedures	*p*
Clinically significant bleeding	2 (3%)	0 (0%)	2 (4%)	0.420
Myocardial injury	0	0	0	Ø
Ischemic stroke	1 (1.5%)	0 (0%)	1 (2%)	0.572

## Data Availability

Data presented in this study were extracted by PR and PA; the raw datasets of this study may be available from the corresponding author upon reasonable request.

## Supplementary Materials

The following supporting information can be downloaded at: https://www.mdpi.com/article/10.3390/cancers16213698/s1, Table S1: Hemodynamic parameters; Table S2: Laboratory values; Figure S1: MAP (in mmHg) and heart rate (in bpm).

## List of Abbreviations

ACT	Activated Clotting Time
AT3	Antithrombin-3
ECC	Extracorporeal Circulation
FFP	Fresh Frozen Plasma
HES	Hydroxyethyl Starch
IJV	Internal Jugular Vein
NE	Norepinephrine
NO	Nitric Oxide
PCC	Prothrombin Complex
RA	Right Atrium
RBC	Red Blood Cell
TACO	Transfusion-Associated Circulatory Overload
TEE	Transesophageal Echocardiography
TRALI	Transfusion-Related Lung Injury
Vaso	Vasopressin
VCI	Vena Cava Inferior
VIS	Vasoactive Inotropic Score
