# Supplementary material for: Peri-Interventional Hemodynamic Management Strategies for Percutaneous Chemosaturation of the Liver in Metastatic Cancer
Source: Cancers (Basel). 2024 Nov 1;16(21):3698. doi: 10.3390/cancers16213698 (PMC11545105; doi:10.3390/cancers16213698)
Supplement: Supplementary file 1 [file cancers-16-03698-s001.zip › cancers-3229932-supplementary.pdf]

Supplements

	Overall<br>n=66 procedures	Group 1 (NE only)<br>n=16 procedures	Group 2 (NE + Vaso)<br>n=50 procedures	p value
**Noradrenaline pre [µg/kg/min]**	0.05 (0.03 – 0.07)	0.03 (0.02 – 0.11)	0.05 (0.03 – 0.07)	0.374
**Noradrenalin during [µg/kg/min]**	0.6 (0.36-1.0)	0.32 (0.25-0.72)	0.71 (0.2-1.0)	0.002
**Noradrenalin post [µg/kg/min]**	0.09 (0.05-0.13)	0.06 (0-0.14)	0.1 (0.06-0.13)	0.063
**Noradrenalin ICU [µg/kg/min]**	0.00 (0.00-0.01)	0 (0-0)	0 (0-0.04)	0.062
**Vasopressin pre**	0 (0-0)	Ø	0 (0-0)	Ø
**Vasopressin during [IU/kg/min]**	0.01 (0.0003-0.0015)	Ø	0.0013 (0.0008-0.0018)	Ø
**Vasopressin post [IU/kg/min]**	0 (0)	Ø	0 (0-0)	Ø
**Vasopressin ICU [IU/kg/min]**	0 (0)	Ø	0 (0-0)	Ø
**VIS pre**	5 (3 – 7.2)	3.8 (2 – 10.8)	5 (3 – 7.2)	0.416
**VIS during**	78.5 (46.6-108)	31.5 (24.5-72)	89 (66.5-114.2)	<0.001
**VIS post**	9.5 (5-13)	6 (0-13.5)	10 (6-13)	0.061
**VIS ICU**	0 (0-0.5)	0 (0-0)	0 (0-3.25)	0.067
**Fluid balance [ml]**	2950 (2300-3988.75)	2340 (1562.5-3850)	3125 (2387.5-3988.75)	0.097
**Crystalloid [ml]**	3500 (3000-4000)	2750 (2000-3875)	3500 (3000-4500)	0.010
**Urine output [ml]**	700 (450-1010)	1000 (387.5-1275)	605 (450-925)	0.161
**HES [ml]**	0 (0-0)	1000 (250-1500)	0 (0-0)	<0.001
**Overall Fluids [ml]**	3500 (3000 – 4500)	3500 (3000 – 4375)	3500 (3000 – 4500)	0.698
**Heart rate pre**	60 (55 – 76.3)	57.5 (50 – 73.8)	60 (60 – 80)	0.095
**Heart rate during**	105 (90-120)	110 (92.5-120)	100 (87.5-120)	0.868
**Heart rate post**	80 (70-90)	75 (61 – 90)	80 (70-90)	0.446
**Heart rate ICU**	80 (70-96)	80 (70-80)	90 (77-100)	0.012
**MAP pre**	80 (75 – 80)	80 (69 – 80)	80 (76.5 – 80)	0.783
**MAP during**	73 (60-81)	70 (52.5-78.75)	73 (62.25-87)	0.270
**MAP after**	80 (75-80)	80 (75-80)	80 (75-80)	0.927
**MAP ICU**	80 (80-86)	80 (80-85)	80 (80-87)	0.572

Suppl. Table S1: Hemodynamic parameters pre, during, post filtration phase and ad admission to the ICU. Data are expressed as Median (interquartile range). NE: Norepinephrine, Vaso: Vasopressin, VIS: Vasoactive-Inotropic-Score, HES: Hydroxyethyl-Starch, MAP: mean arterial pressure

	Overall<br>n = 66 procedures	Group 1 (NE only)<br>n = 16 procedures	Group 2 (NE + Vaso)<br>n = 60 procedures	p value
**Lactate pre [mg/dl]**	10.7 (9 – 15.1)	12 (10.1 – 14.9)	10.6 (8 – 15.3)	0.173
**Lactate during [mg/dl]**	19.9 (15.2-33.9)	23.5 (12.45-36.33)	19.1 (15.45-34.85)	0.818
**Lactate post [mg/dl]**	22.35 (16.67-33.92)	24.25 (18-29.73)	21.25 (15.4-35.9)	0.523
**Lactate ICU [mg/dl]**	15.45 (11.82-24.28)	22.9 (13.65-31.55)	14.45 (11.2-21.3)	0.041
**Creatinine pre [mg/dl]**	0.71 (0.65 – 0.82)	0.78 (0.57 – 0.85)	0.7 (0.67 – 0.79)	0.805
**Creatinine post [mg/dl]**	0.57 (0.49-0.69)	0.55 (0.43-0.71)	0.59 (0.5-0.69)	0.410
**Creatinine day 1 [mg/dl]**	0.71 (0.61-0.81)	0.71 (0.59-0.82)	0.71 (0.61-0.81)	0.742
**Creatinine day 2 [mg/dl]**	0.71 (0.66-0.83)	0.74 (0.51-0.81)	0.71 (0.66-0.8)	0.916
**LDH pre [U/l]**	489 (296-760)	536 (447-829)	470 (288-751)	0.419
**LDH post [U/l]**	411 (259-678)	421 (270-782)	404 (258-678)	0.962
**LDH day 1 [U/l]**	538 (264-1265)	483 (289-1390)	544 (263-1201)	0.876
**LDH day 2 [U/l]**	840 (328-1915)	626 (250-1351)	843 (335-1992)	0.478
**MELD pre**	6 (6 – 6)	6 (6 – 6)	6 (6 – 6)	0.436
**MELD post**	8 (6 – 10)	6 (6 – 8)	8 (7 – 10)	0.001
**GOT pre [U/l]**	40 (25 – 67)	50 (25 – 69)	39 (24 – 64)	0.821
**GOT post [U/l]**	62 (35 – 99)	64 (43 – 97)	60 (33 – 109)	0.510
**GOT day 1 [U/l]**	61 (33 – 184)	57 (33 – 189)	74 (34 – 161)	0.692
**GOT day 2 [U/l]**	68 (35 – 135)	76 (31 – 108)	64 (37 – 142)	0.480
**GPT pre [U/l]**	31 (18 – 49)	38 (17 – 62)	27 (20 – 47)	0.472
**GPT post [U/l]**	36 (26 – 78)	55 (27 – 82)	33 (26 – 78)	0.305
**GPT day 1 [U/l]**	34 (21 – 95)	64 (22 – 108)	33 (21 – 86)	0.474
**GPT day 2 [U/l]**	42 (27 – 92)	67 (32 – 131)	37 (27 – 83)	0.194
**Platelets pre [/nl]**	284 (234 – 384)	259 (199 – 384)	289 (245 – 387)	0.342
**Platelets post [/nl]**	127 (76 – 172)	159 (131 – 242)	113 (69 – 156)	0.022
**Platelets day 1 [/nl]**	121 (81 – 179)	167 (138 – 249)	101 (75 – 150)	0.001
**Platelets day 2 [/nl]**	133 (96 – 175)	158 (121 – 244)	124 (92 – 166)	0.032
**Hb pre [g/dl]**	12 (11.2 – 13.3)	11.6 (10.9 – 13)	12.2 (11.5 – 13.4)	0.278
**Hb post [g/dl]**	10.9 (9.7 – 11.8)	9.6 (8.8 – 11.4)	11.2 (10.1 – 11.9)	0.029
**Hb day 1 [g/dl]**	9.7 (8.8 – 10.8)	9.5 (8.8 – 11.4)	9.7 (8.8 – 10.7)	0.881
**Hb day 2 [g/dl]**	9.7 (9.1 – 11.3)	9.8 (8.8 – 12)	9.7 (9.1 – 11.1)	0.477
**INR pre**	1.01 (0.96 – 1.06)	1.01 (0.97 – 1.1)	1.01 (0.95 – 1.04)	0.337

**INR post**	1.23 (1.16 – 1.38)	1.13 (1.07 – 1.34)	1.26 (1.18 – 1.39)	0.015
**INR day 1**	1.09 (1.04 – 1.17)	1.08 (0.99 – 1.17)	1.09 (1.05 – 1.16)	0.298
**INR day 2**	1.03 (0.99 – 1.06)	1.02 (0.96 – 1.07)	1.03 (0.99 – 1.06)	0.727
**pTT pre [s]**	24 (22.6 – 25.5)	24.5 (23.1 – 25.5)	24 (22.3 – 25.8)	0.765
**pTT post [s]**	27.6 (24 – 114)	26.6 (24.5 – 28.8)	33 (24 – 120)	0.125
**pTT day 1 [s]**	23.5 (23.4 – 27.4)	23.8 (22.6 – 26.5)	25.7 (23.5 – 27.6)	0.134
**pTT day 2 [s]**	23.6 (22.3 – 25.4)	22.4 (21.5 – 24.5)	23.9 (22.5 – 25.7)	0.074
**Fibrinogen post [g/l]**	2.5 (2.14 – 2.69)	2.45 (1.97 – 3.42)	2.5 (2.14 – 2.9)	0.892

Suppl. Table S2: Laboratory values pre/post Chemosaturation and day 1 respectively day 2 after Chemosaturation. Data are expressed as Median (interquartile range). NE: Norepinephrine, Vaso: Vasopressin, MELD: Model of End Stage Liver Disease

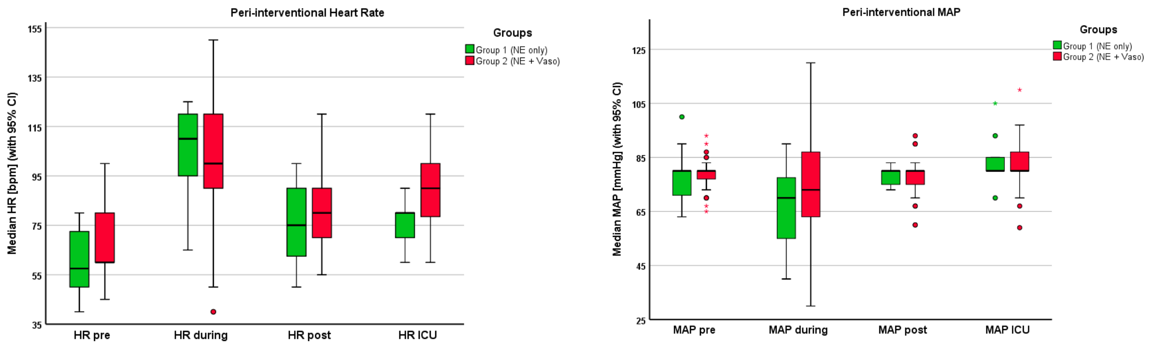

Suppl. Figure S1: MAP (in mmHg) and heart rate (in bpm) pre/during/post filtration phase and at admission to the ICU. MAP: Mean Arterial Pressure, HR: Heart Rate, NE: Norepinephrine, Vaso: Vasopressin, CI: Confidence Interval.
